# Testing the radiation cascade in postglacial radiations of whitefish and their parasites: founder events and host ecology drive parasite evolution

**DOI:** 10.1093/evlett/qrae025

**Published:** 2024-06-19

**Authors:** Jan Brabec, Jérémy Gauthier, Oliver M Selz, Rune Knudsen, Julia Bilat, Nadir Alvarez, Ole Seehausen, Philine G D Feulner, Kim Præbel, Isabel Blasco-Costa

**Affiliations:** Department of Invertebrates, Natural History Museum of Geneva, Geneva, Switzerland; Department of Evolutionary Parasitology, Institute of Parasitology, Biology Centre of the Czech Academy of Sciences, České Budějovice, Czech Republic; Department of Invertebrates, Natural History Museum of Geneva, Geneva, Switzerland; Department of Fish Ecology and Evolution, Centre of Ecology, Evolution and Biogeochemistry (CEEB), Eawag Swiss Federal Institute of Aquatic Science and Technology, Kastanienbaum, Switzerland; Aquatic Restoration and Fisheries section, Federal Office for the Environment (FOEN), Bern, Switzerland; Department of Arctic Biology, The Arctic University of Norway, Tromsø, Norway; Department of Invertebrates, Natural History Museum of Geneva, Geneva, Switzerland; Department of Invertebrates, Natural History Museum of Geneva, Geneva, Switzerland; Department of Genetics and Evolution, University of Geneva, Geneva, Switzerland; Department of Fish Ecology and Evolution, Centre of Ecology, Evolution and Biogeochemistry (CEEB), Eawag Swiss Federal Institute of Aquatic Science and Technology, Kastanienbaum, Switzerland; Division of Aquatic Ecology & Evolution, Institute of Ecology and Evolution, University of Bern, Bern, Switzerland; Department of Fish Ecology and Evolution, Centre of Ecology, Evolution and Biogeochemistry (CEEB), Eawag Swiss Federal Institute of Aquatic Science and Technology, Kastanienbaum, Switzerland; Division of Aquatic Ecology & Evolution, Institute of Ecology and Evolution, University of Bern, Bern, Switzerland; Norwegian College of Fishery Science, UiT The Arctic University of Norway, Tromsø, Norway; Department of Forestry and Wildlife Management, Inland Norway University of Applied Science, Elverum, Norway; Department of Invertebrates, Natural History Museum of Geneva, Geneva, Switzerland; Department of Arctic Biology, The Arctic University of Norway, Tromsø, Norway

**Keywords:** speciation, host repertoire expansion, population genetics, species flocks, RADseq, Platyhelminthes

## Abstract

Reciprocal effects of adaptive radiations on the evolution of interspecific interactions, like parasitism, remain barely explored. We test whether the recent radiations of European whitefish (*Coregonus* spp.) across and within perialpine and subarctic lakes promote its parasite *Proteocephalus fallax* (Platyhelminthes: Cestoda) to undergo host repertoire expansion via opportunity and ecological fitting, or adaptive radiation by specialization. Using de novo genomic data, we examined *P. fallax* differentiation across lakes, within lakes across sympatric host species, and the contributions of host genetics versus host habitat use and trophic preferences. Whitefish intralake radiations prompted parasite host repertoire expansion in all lakes, whereas *P. fallax* differentiation remains incipient among sympatric fish hosts. Whitefish genetic differentiation per se did not explain the genetic differentiation among its parasite populations, ruling out codivergence with the host. Instead, incipient parasite differentiation was driven by whitefish phenotypic radiation in trophic preferences and habitat use in an arena of parasite opportunity and ecological fitting to utilize resources from emerging hosts. Whilst the whitefish radiation provides a substrate for the parasite to differentiate along the same water-depth ecological axis as *Coregonus* spp., the role of the intermediate hosts in parasite speciation may be overlooked. Parasite multiple-level ecological fitting to both fish and crustacean intermediate hosts resources may be responsible for parasite population substructure in *Coregonus* spp. We propose parasites’ delayed arrival was key to the initial burst of postglacial intralake whitefish diversification, followed by opportunistic tapeworm host repertoire expansion and a delayed nonadaptive radiation cascade of incipient tapeworm differentiation. At the geographical scale, dispersal, founder events, and genetic drift following colonization of spatially heterogeneous landscapes drove strong parasite differentiation. We argue that these microevolutionary processes result in the mirroring of host–parasite phylogenies through phylogenetic tracking at macroevolutionary and geographical scales.

## Teaser

Adaptive species radiations generate stunning diversity of organisms and have broad effects on ecosystems. Yet, we lack understanding of how species radiations affect the evolution of its symbionts through radiation cascades. Diverse habitats support diverse communities of free-living species. Likewise, bursts of host diversities will provide resource-rich habitats favoring symbiont species radiations by specialization, or conversely fostering symbiont expansion into emerging hosts. We tested this hypothesis using a parasitic tapeworm that infects European whitefish, a model system of recent postglacial radiation. Whitefish radiation within lakes provided the substrate for the parasite to differentiate along the same benthic–pelagic ecological axis of lakes, but most likely by specializing in different crustacean intermediate hosts rather than by adaptation to the fish host. Dispersal, founder events, and genetic drift during colonization of heterogeneous landscapes drive parasite differentiation spatially. These microevolutionary processes may be responsible for the mirroring of host–parasite evolutionary histories at the macroevolutionary scale.

## Introduction

Adaptive radiations in which organisms diversify rapidly into multitudes of new species have occurred repeatedly throughout the history of life on Earth (Gillespie et al., 2020), often in response to environmental changes that make new resources or niches available (Schluter, 2000). This rise of diversity in ecological roles has broad ecosystem effects (Lundsgaard-Hansen et al., 2017) and may propagate to interacting species resulting in an adaptive radiation cascade (Brodersen et al., 2018; Walsh et al., 2012). Yet, evidence is still scarce, and the mechanisms that drive the evolution of symbiotic and interactive species in radiation cascades remain untested. Analogous to how diverse habitats support diverse communities of free-living species (Lawton, 1983), host diversity can foster parasite community diversity (Johnson et al., 2016; Kamiya et al., 2014) through providing variable and resource-rich habitats promoting symbiont species radiations by specialization (e.g., Abrahamson & Blair, 2008; Feder & Forbes, 2010; Lutzoni et al., 2018; Vanhove et al., 2015). Alternatively, the Stockholm Paradigm (SP), a framework for understanding the evolution of diversity and interspecific associations, postulates that external perturbations changing the environmental conditions of the organisms (hosts or symbionts) will foster them to oscillate from exploitation (specialization) into the exploration (generalization) mode within the limits of their capacity and opportunities (Agosta, 2023; Agosta & Brooks, 2020; Brooks et al., 2019; Nylin et al., 2018). Thus, with hosts undergoing speciation, symbionts can expand their host repertoire (e.g., Gobbin et al., 2021; Kmentová et al., 2020) as a result of ecological opportunity and ecological fitting in sloppy fitness space (Agosta et al., 2010; Braga et al., 2018; Hoberg & Brooks, 2008). Since most symbiont groups remain unstudied (Althoff et al., 2014; Clayton et al., 2015; Ricklefs et al., 2014), more insights are needed before inferring general trends on the effect of host radiations on their symbionts.

Amongst symbionts, parasites are ubiquitous and key components of their host environment (Poulin & Morand, 2004), with whom they engage in reciprocal selection dynamics (Thompson, 2005) and other evolutionary processes that foster micro- and macroevolutionary change (Clayton et al., 2015; Paterson et al., 2010). Metazoan parasite biological characteristics influence their population parameters and/or evolutionary rates in ways uncharacteristic of free-living animals, including their reproductive strategies (e.g., hermaphroditism with self-fertilization and outcrossing, asexual reproduction, or both); short generation times; highly fragmented or isolated populations of endoparasites (each generation reproduces inside a single host individual, regardless of the number of available hosts in the habitat), tendency to seasonal fluctuations due to host death or colonization, resulting in local population extinctions and founder events, respectively, which lead to low effective population sizes and increased mutation fixation rates, favoring their rapid divergence (Huyse et al., 2005). Despite their great potential for speciation research, only a handful of parasitic helminth taxa have been investigated in the context of host radiations (e.g., Beveridge et al., 2002; Rahmouni et al., 2022; Vanhove et al., 2015). These studies are often limited to investigation of parasites with direct (one-host) life cycles, relatively low complexity models allowing to control for important biological attributes (e.g., dispersal). The level of interdependency in parasites with multi-host strategies (e.g., in Rotifera, most Platyhelminthes, Cnidaria, some Nematoda) thus remains unclear (Blasco-Costa & Poulin, 2013; Mazé-Guilmo et al., 2016). Using multiple sequential hosts through the parasite’s development provides heterogeneous selection regimes (Braga et al., 2018; Poulin, 2011) and prolongs generation times—the passage to a downstream host can take even decades (e.g., Curtis, 2003; Moore, 1987)—and thus, parasite’s evolutionary rates might approximate those of its host, contrary to the elevated rates of one-host parasites (e.g., Hafner et al., 1994). Furthermore, most diversification studies of host–parasite associations spanning several million years applied macroevolutionary approaches that can undergo time-dependent bias in the estimation of evolutionary rates (reviewed in Ho et al. (2011)), postspeciation evolution and geological factors [extinctions, bottlenecks (e.g., Drábková et al. (2019)], which can obscure the inferences of diversification/speciation processes. Instead, population genetic studies on parasite recent postglacial differentiation are still rare (Nieberding et al., 2005; Perrot-Minnot et al., 2018), and often based on single mitochondrial markers [but see Nazarizadeh et al. (2023)]. The use of host–parasite models that differentiated more recently may avoid some of these shortcomings and shed new light on the speciation process in parasites.

The European whitefish (*Coregonus lavaretus* species complex) is a model system for studying rapid speciation and adaptive radiation (Bernatchez, 2004; Hudson et al., 2007) having undergone a complex history of parallel diversifications in large and deep lakes across the Northern Hemisphere after the last glacial maximum, ca 10,000–15,000 BP [reviewed in Hudson et al. (2007)]. Genetically distinct whitefish lineages recolonized Europe from different glacial refugia (Crotti et al., 2021; Hudson et al., 2011; Østbye et al., 2005; Rougeux et al., 2019). Thereafter, species flocks formed through a combination of geographically sympatric and allopatric speciation in high-latitude lakes (Østbye et al., 2005; Praebel et al., 2013), and in perialpine lakes mostly through geographically sympatric speciation from an ancestral hybrid population (Hudson et al., 2011). Intralake diversification occurs along the water-depth axis and is often resource driven, with further subdivision into the littoral and the profundal habitats occasionally (Ingram et al., 2012; Siwertsson et al., 2013). Perialpine European whitefish are monophyletic with respect to their closest relatives outside the region (Hudson et al., 2011). Thus, the lake colonization and sympatric diversification of European whitefish represent a uniquely defined recent radiation model system for parasite evolutionary ecology research.

Tapeworms (Platyhelminthes) are obligatory endoparasites that complete a generation through sequential colonization of several hosts. They are thus suitable models to investigate complex host–parasite associations within the framework of the SP and to test for the adaptive radiation cascade. This study examines the patterns of differentiation within *Proteocephalus fallax* La Rue, 1911, a specialist tapeworm parasitizing European whitefish, and recently recognized as a species separate from *Proteocephalus longicollis* (Zeder, 1800) (Brabec et al., 2023). Until now, *P. longicollis* sensu lato (*s.l.*) comprised numerous synonyms of species previously described from various salmoniform fishes, including species of *Coregonus*, *Oncorhynchus*, *Salmo*, *Salvelinus*, and *Thymallus* (Hanzelová & Scholz, 1999; Scholz & Hanzelová, 1998). *Proteocephalus longicolliss.l.* (including *P. fallax*) circulates between two aquatic hosts, an invertebrate (several copepod species) and a vertebrate (salmoniform fishes), whereas its short-lived free-living larval stage disperses by flotation (Scholz, 1999). Thus, parasite’s dispersal is strongly linked to its aquatic hosts. Development of *P*. *longicolliss.l.* follows seasonal cycles influenced by abiotic factors (mainly water temperature) and its life cycle takes 1 year to complete (Chubb, 1982). Of all whitefish parasites, its relatively large size offers sufficient DNA quantities to perform state-of-the-art genomic methods to investigate recent divergence with sufficient detail.

**Figure 1.**
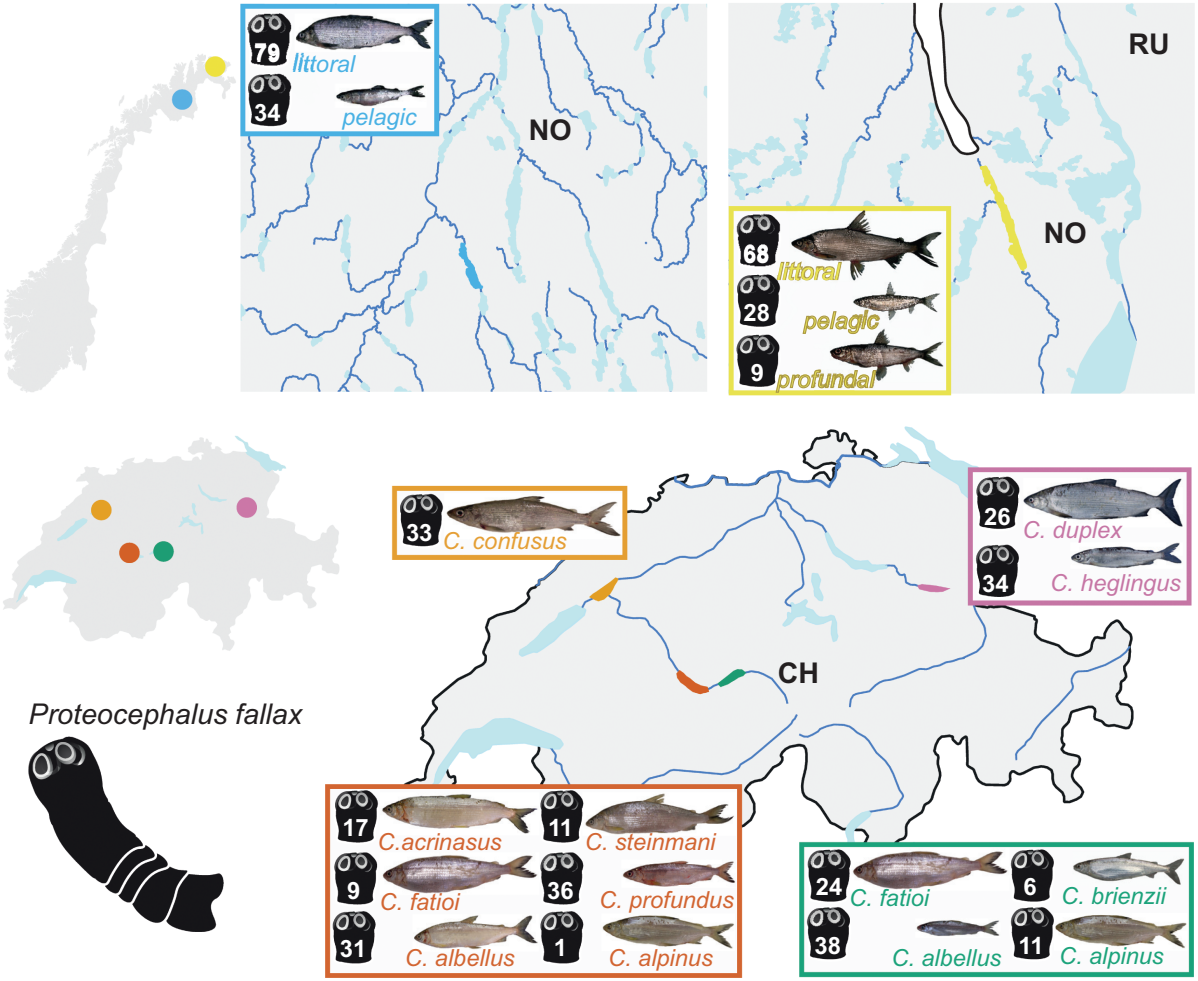
Maps of the two regions studied, Switzerland and northern Norway (insets on the left), with lakes sampled in each region color-coded (enlarged maps: Suohpatjávri, blue; Langfjordvatn, yellow; Bienne, orange; Walen, pink; Thun, dark orange; Brienz, dark green). European whitefish species/ecotypes illustrations are accompanied by black parasite silhouettes showing the number of *Proteocephalus fallax* specimens genotyped from each host species. Two-letter country codes for reference.

The recent establishment and in situ evolution of the whitefish–*P. fallax* associations within postglacial lakes make this system an excellent natural experiment suitable to test the adaptive radiation cascade hypothesis and elucidate the mechanisms promoting or preventing parasite differentiation within the SP framework, minimizing large-timescale confounding factors. The novelty lies in investigating population-level differentiation of a complex life cycle parasite in a recently radiated host. We predict that the pressures exerted by the intermediated host and the environment might outweigh the influence of definitive host radiation in this multi-host–parasite differentiation. Using double digest restriction-site associated DNA sequencing (ddRAD) to obtain variable markers for a postglacial radiation scenario, we seek to resolve whether the paradigm “host diversity begets parasite diversity” holds true in the context of whitefish and their tapeworms, by analyzing six replicate lake radiations in two geographically separated areas (Figure 1). Or Conversely, whether the whitefish diversification fosters *P. fallax* host range expansion by ecological fitting. Our study disentangles the contributions of the fish host genetic and phenotypic diversification (in trophic ecology and habitat use) in driving parasite differentiation. We characterize *P. fallax* population genomic structure at multiple scales: within lakes across sympatric *Coregonus* species, and across lakes at regional and continental scales. The study also investigates how lake abiotic characteristics relate to parasite population genetic parameters to shed light on environmental influences on parasite diversity.

## Materials and methods

### Fish sampling, identification, and infection statistics

European whitefish were collected from four perialpine lakes in Switzerland and two subarctic lakes in northern Norway (Figure 1) by fishermen or through scientific fishing (permits ZH128/15 and Sak2015/266) between July and December 2017. Fin clips were preserved in ethanol for genotyping and fish species/ecotype identification was done based on genetic results and morphology (see details in Supplementary material). Worms were extracted from the fish intestine and preserved in ethanol.

### Parasite whole genome sequencing and assembly

The implementation of genomic analyses of small organisms, like parasitic helminths, is hampered by the limited amount of tissue for obtaining sufficient high-molecular-weight genomic DNA (gDNA) and the risk of contamination with host DNA. *Proteocephalus fallax* genomic data were generated from a single specimen originating from Lake Geneva (Switzerland) using MinION (Oxford Nanopore Technologies) and NovaSeq 6000 (Illumina) platforms (see Supplementary material for details). Genome assembly was performed on Nanopore raw reads using FLYE v2.8.1(Kolmogorov et al., 2019) with a polishing step integrating Illumina reads and using ntHits and ntEdit (Warren et al., 2019). Genome completeness was assessed using BUSCO v3 (Simão et al., 2015; Warren et al., 2019) with the Metazoa database (metazoa_odb9) including 978 genes.

### Parasite DNA isolation, library preparation, and ddRAD sequencing

To extract DNA from small tapeworm individuals and limit sample cross-contamination risks, we adapted the Monarch PCR & DNA Cleanup kit protocol (New England Biolabs). We used 100 μL TL buffer of the E.Z.N.A. Tissue DNA Kit (Omega Bio-tek) and 80 μg Proteinase K (Sigma-Aldrich) to carry out 1-hour tissue lysis at 56 °C followed by manual Monarch PCR & DNA Cleanup following the manufacturer’s protocol (with a volume ratio of binding buffer:sample of 2:1), finalized by two subsequent 7 μl preheated water elutions. A total of 875 gDNA single worm extractions were performed and quantified with Quant-iT PicoGreen dsDNA Assay Kit on Hidex Sense microplate reader, of which 497 produced sufficient DNA amounts and were used for library construction (including 48 technical duplicates).

Our ddRAD library construction protocol followed Peterson et al. (2012), adapted to suggestions of Mastretta-Yanes et al. (2015); (see the step-by-step protocol in Supplementary material). Due to the lack of both prior ddRAD studies and a reference genome of a related tapeworm, we first empirically evaluated the performance of several restriction enzyme pairs (Supplementary material), which led to the selection of two relatively frequent cutters NlaIII and MseI endonucleases as the pair producing preferable gDNA digestion profile. Sixty nanograms gDNA (minimum concentration of 10 ng/μl) was targeted as the starting amount for gDNA double digestion, followed by ligation of adaptors tagged with 24 different barcodes, 20 cycles of PCR amplification with reverse primers tagged with 16 different indexes, and size selection retaining fragments of 320–500 bp. Two equimolar pools of 384 ddRAD libraries each were sequenced at the Lausanne Genomics Technologies Facility using 6 lanes of Illumina HiSeq 2500 and 150 bp paired-end reads.

### Reads cleaning, mapping, and SNP calling

Raw ddRAD reads were demultiplexed and trimmed with the first step of ipyrad (Eaton & Overcast, 2020) allowing for one mismatch in barcodes. Read quality was assessed using FastQC (Andrews, 2010). Trimmed reads were mapped to the reference genome using bwa-mem (Li & Durbin, 2009), and reads with mapping quality <20 removed using samtools (Li et al., 2009) to avoid reads with multiple mappings and putative paralogs. Indel realignment was performed using the Picard toolkit (http://broadinstitute.github.io/picard) and IndelRealigner from GATK4 (Van der Auwera et al., 2013). SNP calling was performed using HaplotypeCaller algorithm from GATK4 (Van der Auwera & O'Connor 2020) combining all samples to improve variant detection. SNPs were extracted using VCFTOOLS (Danecek et al., 2011) to keep bi-allelic SNPs with a calling quality >20 and shared by at least 50% of the samples.

### Population genetic analysis

SNPs were filtered to keep only a single SNP per ddRAD locus (spaced by at least 200 bp) to minimize the effects of linkage disequilibrium within ddRAD loci. We performed a principal component analysis implemented in the *adegenet* R package (Jombart & Ahmed, 2011) and a Bayesian admixture analysis using STRUCTURE. We tested K from 1 to 10 with 3 independent Markov chains each, using 200,000 steps, including 10,000 burn-in steps, and verified chains convergence to a stable posterior distribution in each run. The most likely number of clusters was identified using Evanno’s method (Evanno et al., 2005) implemented in Structure Harvester (Earl & vonHoldt, 2012). Genome-wide weighted genetic differentiation (F_ST_) was estimated using Weir and Cockerham’s method (Weir & Cockerham, 1984) implemented in VCFTOOLS. Genetic diversity, heterozygosity, allelic richness, and inbreeding coefficient were estimated using the *hierfstat* R package (Goudet, 2005). Effective population sizes (Ne) were estimated for each lake using the bias-corrected measure of linkage disequilibrium (Waples & Do, 2010) implemented in NeEstimator v.2.1 (Do et al., 2014) with a minor allele frequency cutoff of 0.05.

### Testing *Proteocephalus* differentiation in replicated whitefish radiations

Pairwise F_ST_ were estimated among *Proteocephalus* populations from distinct sympatric *Coregonus* spp./ecotypes for each lake, using the *hierfstat* R package (Goudet, 2005). For each comparison, 999 random permutations were performed to estimate a random F_ST_ distribution followed by a Monte-Carlo test to assess whether the observed F_ST_ value was significantly higher than the random distribution. A Bonferroni correction of the *p*-values was applied to correct for multiple comparisons. Pairwise F_ST_ among *Coregonus* species/ecotypes were estimated in GenAlEx (Peakall & Smouse, 2012). Differentiation of *Proteocephalus* in relation to the genetic differentiation of *Coregonus* spp. was examined by regressing pairwise F_ST_ values of *P*. *fallax* populations from sympatric hosts to those of sympatric *Coregonus* spp./ecotypes using a generalized linear model with a quasibinomial error distribution and a logit link function, implemented in the *stats* R package.

### Spatial and environmental effects on genetic differentiation and diversity indexes

We performed a distance-based redundancy analysis (dbRDA) integrating geographical variables, four quantitative variables on lake characteristics (lake surface, maximum depth, oxygenated lake depth and past highest phosphorous levels), and two quantitative variables on host fish ecology transformed in dummy variables, that is, fish resource preferences (planktivorous = 0, mixed feeding = 1, and benthivorous = 2) and fish habitat occupation (profundal = 0, wide distributed = 1, and shallow = 2), in an individual-based approach (see Laura Benestan https://github.com/laurabenestan/db-RDA-and-db-MEM). In brief, we first created spatial variables using Moran Eigenvector’s Maps (MEMs) implemented in the *adespatial* R package (Dray et al., 2023). Second, a principal coordinates analysis (PCoA) was performed on the Euclidean genetic distances based on the genotypes of each sample. Finally, a global dbRDA was applied by integrating ecological factors as additional variables to spatial components, and an ANOVA with 1,000 permutations was performed to assess the significance of each variable within the model using the *vegan* R package (Oksanen et al., 2020). The relationship between the three current environmental lake characteristics mentioned above and the parasite genetic diversity indices was assessed using Spearman’s correlation test for each variable independently using the *stats* R package. The R computing environment v. 4.1.2 was used (R Development Core Team, 2021).

## Results

### Population census data of *P. fallax* among sympatric whitefish and lakes

Tapeworm prevalence (percentage of fish infected among all examined) was high in all lakes, spanning 86–99%, the lowest in Langfjordvatn coinciding with the lowest mean intensity of infection (average number of worms per infected fish), and the highest in Brienz (Table 1). *Proteocephalus fallax* highest mean intensities of infection were achieved in lakes Bienne and Brienz (range: 1–1,000 individuals; Table 1). Among the perialpine hosts, the highest mean intensities were found in *C*. *confusus* (234 ± 52) from lake Bienne, *C*. *fatioi* (256 ± 72) and *C*. *albellus* (139 ± 23) from lake Thun, and *C*. *brienzii* (121 ± 42), *C*. *fatioi* (102 ± 14) and *C*. *albellus* (94 ± 17) from lake Brienz (Supplementary Table S1), all species typically considered pelagic feeders. In the subarctic lakes, the pelagic ecotypes also carried the highest mean intensities. All other European whitefish species/ecotypes harbored lower mean intensities of infection (6–73 individuals), so that they carry a small proportion of the *P*. *fallax* population in each lake.

**Table 1.** Summary statistics of genetic diversity per population using 8,072 SNPs and infection parameters per lake. N *P*. *fallax*: sample size of parasites, N SNPs: number of SNPs retained per lake, CIs: confidence intervals, Mean Intensity ± S.E.: a measure of population census size indicating the average number of *P*. *fallax* specimens and standard error per infected fish, Prevalence of infection: percentage of fish infected from all whitefish examined at each lake, also a measure of population census size.

	Perialpine lakes				Subarctic lakes	
	Bienne	Brienz	Thun	Walen	Langfjordvatn	Suohpatjávri
N *P*. *fallax*	33	93	110	60	105	113
N SNPs	34,843	4,758	33,488	10,152	12,375	11,611
Allelic richness (Ar)	1.34	1.54	1.63	1.44	1.28	1.11
Genetic diversity (Hs)	0.15	0.16	0.16	0.16	0.15	0.11
Observed heterozygosity (Ho)	0.07	0.06	0.09	0.05	0.05	0.04
Inbreeding coefficient (Fis)	0.56	0.62	0.46	0.65	0.66	0.63
Effective Population size (Ne)	910	1,932	627	450	1,253	1,170
95% CIs	692−1,329	780−inf	600−657	352−622	954−1,821	862–1,814
Mean intensity ± SE	234 ± 52	93 ± 10	68 ± 8	52 ± 7	20 ± 4	68 ± 10
Intensity of infection (range)	3−1,000	1 − 749	1 − 520	1−300	1−255	1–429
Prevalence of infection (%)	91	99	92	98	86	93

### De novo genome assembly of *P. fallax* from Lake Geneva (Switzerland)

The Oxford Nanopore run produced 1,680,336 reads with a mean length of 2,697.96 bp and the Illumina sequencing produced 345,501,790 reads and a total of 51.82 Gbp (Table 2). The de novo assembly strategy of the Nanopore reads followed by polishing with Illumina reads yielded the first genome for a representative of the cestode order Onchoproteocephalidea. It comprised 4,060 scaffolds with a scaffold N50 of 419,724 bp and a total assembly length of 131,602,485 bp which is comparable to the k-mer estimate performed on Illumina raw reads (110,347,222 bp; see Table 2). BUSCO metrics showed moderate genome completeness (56.5% complete BUSCO; Table 2) as expected for a nonmodel flatworm representative. For comparison, the same BUSCO analysis performed on the chromosome-level reference genome assembly of *Hymenolepis microstoma* (GCA_000469805.3) resulted in 65.7% of complete BUSCO. Illumina reads mapping on the final genome was high, 98.08%, suggesting good completeness of the genome assembly.

**Table 2.** Reference genome statistics on raw data, genome size estimations, genome assembly, and completeness using BUSCO.

Statistics	Illumina	Nanopore
**Raw data**		
Mean read length (sd)	150	2,697.96
# reads	345,501,790	1,680,336
sum (Gb)	51.82	4.53
**Genome size estimation**		
Het	0.0092	
err	0.0037	
genome size	110,347,222	
fit	2.03	
**Assembly statistics**		
# Scaffolds	4,060	
N50 scaffold	419.724	
Mean scaffold size	32.414	
Longest scaffold	3,708,885	
%N	0.0075	
GC content (%)	43.28	
Total length	131,602,485	
**BUSCO results (%)**		
Complete and single-copy BUSCOs	55.2	
Complete and duplicate BUSCOs	1.3	
Fragmented BUSCOs	10.3	
Missing BUSCOs	33.2	

### Genomic descriptors of *Proteocephalus* populations and structure across lakes

A total of 1,283,228,366 quality-trimmed sequence reads were obtained providing an average of 2,496,553 ± 642,993 reads per individual. The mapping step to the newly generated reference genome resulted in an average of 95.27 ± 7.26% mapped reads before cleaning and 72.52 ± 6.78% after cleaning (Supplementary Table S2). The SNP calling identified 8,072 bi-allelic SNPs shared by at least 50% of the samples and an average of 4,495 ± 786 SNPs per sample. A second SNP set was generated keeping only perialpine samples resulting in 26,738 SNPs shared by at least 50% of the perialpine samples. To perform genetic structure analyses, one SNP every 200 bp along the genome was retained, resulting in 2,002 SNP from the dataset generated on all samples and 6,121 SNP from the perialpine dataset. Finally, SNP sets were also generated within each lake using the same parameters as above (Supplementary Table S2).

Based on 8,072 SNPs shared among all samples, *P*. *fallax* from the subarctic lake Suohpatjávri showed lower observed heterozygosity (H_O_), gene diversity (H_S_), and allelic richness (Ar) values, despite its higher mean census population size (approximated from the prevalence and mean intensity of infection data, Table 1) relative to Langfjordvatn. The highest H_O_, H_S_, and Ar values were detected in the perialpine region, in Thun (Table 1), which is also the lake with the highest whitefish species richness (six *Coregonus* spp.). The inbreeding coefficient (F_IS_) was the highest in Langfjordvatn coinciding with the lowest mean census population size of the subarctic lakes, and the lowest in Thun having the highest mean census population sizes in the perialpine lakes. On the contrary, effective population sizes (Ne) were the largest in the subarctic lakes and the perialpine lake Brienz, whereas the same lakes had low values of H_O_ and high F_IS_. Walen showed the lowest Ne coinciding with a highly inbred population and the smallest perialpine census population size (Table 1).

**Figure 2.**
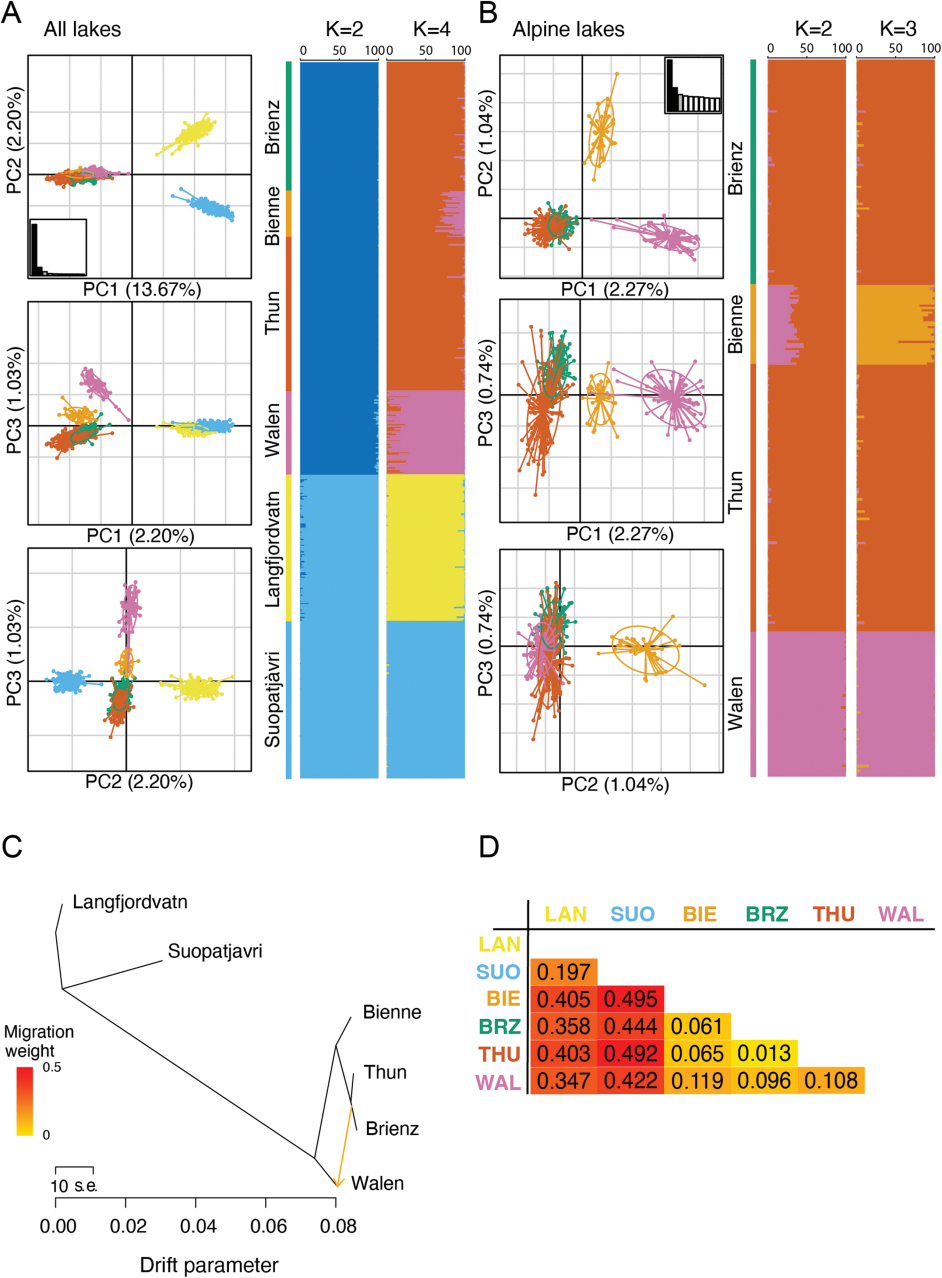
*Proteocephalus fallax* population genetic structure across (A) all lakes based on 2,002 shared SNPs, and (B) perialpine lakes based on 6,121 shared SNPs. The best number of genetic clusters (K) is given for each subset. (C) Phylogenetic network of the historical relationships among the populations inferred by Treemix. Putative gene flow is identified by an arrow pointing in the direction of the recipient population and colored proportionally to the gene flow intensity. (D) Pairwise F_ST_ matrix for all population comparisons. Colors are proportional to the degree of divergence and correspond to F_ST_ values.

Strong genetic structure was detected between the *Proteocephalus* populations from the subarctic and perialpine regions with clearly separated clusters in the PCA and STRUCTURE plots (Figure 2A, K = 2), and F_ST_ values above 0.35 (Figure 2D). Differentiation between populations in the two subarctic lakes was almost as strong as between regions, despite their geographic proximity (Figure 2A,D, K = 4). In the perialpine region, three distinct populations were detected in the lakes Walen, Bienne, and Thun and Brienz (Figure 2B, K = 3). The genetic relationships inferred by the TreeMix analysis (Figure 2C) supported the patterns of genetic differentiation described above. Furthermore, individuals from Thun and Brienz shared a common ancestry and formed a single genetic cluster (F_ST_ = 0.01) (Figure 2C,D).

### *Proteocephalus fallax* differentiation among hosts in replicated whitefish radiations

Genetic structure of parasite populations infecting sympatric whitefish species was overall weak, but significant in most lakes (pairwise F_ST_, Table 3A). Replicated patterns of differentiation appeared between parasite populations from the littoral and pelagic European whitefish ecotypes in Suohpatjávri, and their equivalents, the shallow/littoral and the deep/pelagic in Walen. In the perialpine region, parasite differentiation was common in the most host-rich lake Thun but less in Brienz, with six and four *Coregonus* spp., respectively (Table 3A). In Brienz, only the parasite populations associated with *C*. *brienzii* and *C*. *albellus* showed a significant level of differentiation.

**Table 3.** Comparisons of *P*. *fallax* population genetic structure (F_ST_) amongst A) sympatric fish species of each lake, B) the host habitat use, and C) the host trophic preference.

Lake	Comparison	Parasite F_ST_	*p*-value
**(A)**	**By sympatric fish species**		
Brienz	*C. brienzii* (6) vs. *C. fatioi* (24)	0.017	.051
	*C. brienzii* (6) vs. *C. albellus* (38)	0.026	.007*
	*C. brienzii* (6) vs. *C. alpinus* (11)	0.022	.043
	*C. fatioi* (24) vs. *C. albellus* (38)	0.004	.074
	*C. fatioi* (24) vs. *C. alpinus* (11)	0.010	.031
	*C. albellus* (38) vs. *C. alpinus* (11)	0.009	.042
Langfjordvatn	pelagic (28) vs. littoral (68)	0.003	.047
	pelagic (28) vs. profundal (9)	0.006	.096
	littoral (68) vs. profundal (9)	0.005	.078
Suohpatjávri	littoral (79) vs. pelagic (34)	0.003	.012*
Thun	*C. acrinasus* (17) vs. *C. albellus* (31)	0.002	.121
	*C. acrinasus* (17) vs. *C. profundus* (36)	0.002	.135
	*C. acrinasus* (17) vs. *C. steinmanni* (11)	0.006	.020
	*C. acrinasus* (17) vs. *C. fatioi* (9)	0.014	.001**
	*C. albellus* (31) vs. *C. profundus* (36)	0.004	.001**
	*C. albellus* (31) vs. *C. steinmanni* (11)	0.005	.021
	*C. albellus* (31) vs. *C. fatioi* (9)	0.011	.003*
	*C. profundus* (36) vs. *C. steinmanni* (11)	0.006	.013*
	*C. profundus* (36) vs. *C. fatioi* (9)	0.014	.001**
	*C. steinmanni* (11) vs. *C. fatioi* (9)	0.020	.001**
Walen	*C. duplex* (26) vs. *C. heglingus* (34)	0.006	.003**
**(B)**	**By host habitat use**		
Brienz	shallow (11) vs. wide distribution (68)	0.009	.030*
	shallow (28) vs. wide distribution (68)	0.003	.037
Langfjordvatn	shallow (28) vs. profundal (9)	0.006	.105
	wide distribution (68) vs. profundal (9)	0.005	.083
Suohpatjávri	shallow (34) vs. wide distribution (79)	0.003	.014*
	shallow (18) vs. wide distribution (51)	0.003	.050
Thun	shallow (18) vs. profundal (36)	0.002	.098
	wide distribution (51) vs. profundal (36)	0.005	.001**
Walen	shallow (26) vs. wide distribution (34)	0.006	.005**
**(C)**	**By host trophic preference**		
	mix feeding (30) vs. planktivorous (38)	0.004	.044
Brienz	mix feeding (30) vs. benthivorous (11)	0.010	.023*
	planktivorous (38) vs. benthivorous (11)	0.009	.030
Langfjordvatn	benthivorous (77) vs. planktivorous (28)	0.003	.038*
Suohpatjávri	benthivorous (79) vs. planktivorous (34)	0.003	.014*
	mix feeding (28) vs. planktivorous (40)	0.003	.010*
Thun	mix feeding (28) vs. benthivorous (37)	0.003	.024*
	benthivorous (37) vs. planktivorous (40)	0.005	.001**
Walen	benthivorous (26) vs. planktivorous (34)	0.006	.006**

*p*-Values were estimated by performing 999 permutation sets and observed values were compared to a random distribution using a Monte-Carlo test. Significant values (*) indicate observed values were higher than random distribution and *p*-values were adjusted with Bonferroni correction when more than one comparison in a lake. The *P*. *fallax* population from the single host species in Bienne and the single-genotyped parasite specimen from *C*. *alpinus* in Thun were excluded from these comparisons, as well as the *P*. *fallax* population from the single host species in Bienne. Numbers of genotyped *P*. *fallax* specimens are indicated in parentheses.

**Figure 3.**
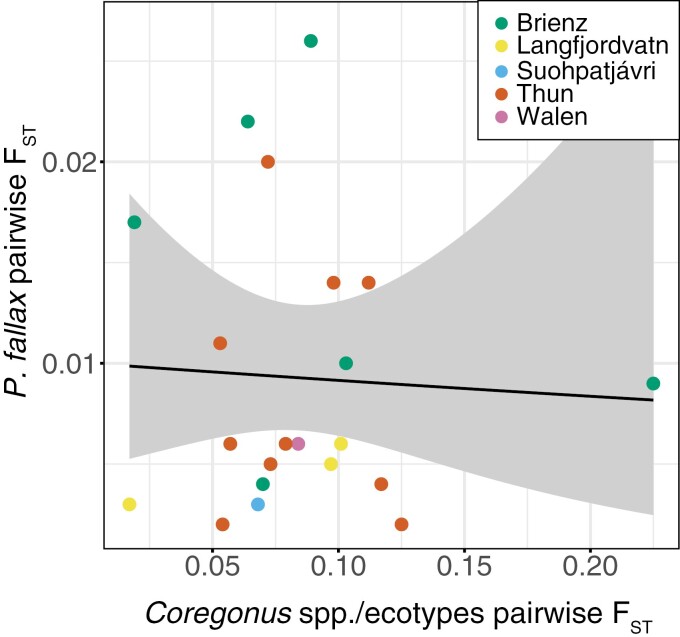
Parasite differentiation was unrelated to the level of differentiation between its hosts within lakes. Regression plot of pairwise F_ST_ estimates between *Proteocephalus fallax* populations in sympatric *Coregonus* hosts (based on SNP data) and pairwise F_ST_ between sympatric *Coregonus* species/ecotypes (based on microsatellite data). Data points are colored according to lakes.

The correlation between *P*. *fallax* population differentiation among sympatric fish host species/ecotypes in each lake and the genetic differentiation between the pairs of sympatric *Coregonus* species/ecotypes was slightly negative (Figure 3), but nonsignificant (GLM_FishFST_: *F*_1,19_ = 0.049, *p *= .830). In other words, host diversification did not explain the differentiation of its parasite populations, for which genetically well-differentiated host species/ecotypes harbor rather genetically weakly differentiated *Proteocephalus* populations. The same pattern emerged after excluding the more differentiated *C*. *alpinus*/*C*. *albellus* pairwise comparison from Lake Brienz (GLM_FishFST_: *F*_1,18_ = 0.098, *p *= .790).

### The contribution of host ecology to *P. fallax* differentiation across replicated radiations

Host ecology played a relevant role in acquiring divergent *P*. *fallax* populations (Table 3B,C). The comparisons among sympatric *Coregonus* spp./ecotypes with distinct habitat use showed that European whitefish species specialized on shallow waters versus those more widespread in the water column exhibit different *P*. *fallax* subpopulations in Suohpatjávri, Brienz, and Walen, whereas in the more host species-rich lake of Thun the differentiation within *P*. *fallax* occurs between the whitefish species groups with wide distributions in the water column and the profundal specialist, *C*. *profundus* (Table 3B). Comparisons of *P*. *fallax* between sympatric European whitefish groups with different trophic preferences revealed in almost all cases significant differentiation, with slightly higher differentiation between *P*. *fallax* in whitefish with benthivorous versus planktivorous feeding strategies (Table 3C). Thus, the intralake differentiation of the subpopulations of this trophically transmitted parasite in the definitive hosts is associated with phenotypic diversification of *Coregonus* spp. in habitat segregation and dietary traits.

### Spatial environmental associations and abiotic correlations

The distance-based redundancy analysis revealed that the spatial component explained most of the genetic variance across Europe (*R*^2^ = .234, *p* = .002). The contribution of the fish host resource preference and habitat occupation were significant, although much lower (*R*^2^ = .039, *p* = .004 and *R*^2^ = .004, *p* = .002, respectively).

Lake physicochemical characteristics, in particular lake maximal and oxygenation depths showed a significant positive correlation with allelic richness (*R*^2^ = .940, *p* = .017; *R*^2^ = .890, *p* = 0.033, respectively) and genetic diversity of *P*. *fallax* (*R*^2^ = .930, *p* = .008; *R*^2^ = .930, *p* = .008), the latter mostly driven by the values in Suohpatjávri (Supplementary Figure S1). Observed heterozygosity showed a strong positive correlation with lake surface area only (*R*^2^ = .990, *p* = .000). Neither allelic richness nor genetic diversity correlated with the number of samples examined or the number of SNPs per lake (Supplementary Figure S2).

## Discussion

Our study showed that populations of the tapeworm *P*. *fallax* were strongly differentiated spatially among lakes, between, and within the perialpine and subarctic regions. Genomic differentiation within lakes was weak, albeit significant, across replicated radiations of European whitefish in both the Alps and the subarctic. Thus, the European whitefish radiation provided a substrate for the parasites to radiate too, which supports the ‘radiation cascade’ hypothesis, although parasite differentiation was independent from the genetic differentiation of the fish hosts. In sympatry, European whitefish trophic preferences and habitat use had weak, but significant and replicated effects on the differentiation among parasite populations in all lakes. Between lakes, allelic richness and parasite genetic diversity significantly correlated with lake maximum depth and lake oxygenation level, but only heterozygosity correlated with lake surface area. Altogether, our results inform about the mode of evolution of an endoparasitic platyhelminth representative and indicate that *P*. *fallax* differentiated spatially in the last 10,000–15,000 years, likely caused by the distinct demographic history of postglacial colonization of the lakes together with its fish hosts. However, genetic differentiation in the tapeworm was constrained in sympatry, being structured along the same water-depth ecological axis of lakes as its *Coregonus* hosts differentiate, but differentiation remains incipient when compared with the rapid genetic and phenotypic diversification of the fish hosts.

### Shared evolutionary history of *P. fallax* and *Coregonus* at the continental and regional scales

The comparison of phylogeographic patterns of hosts and symbionts can help elucidate colonization hypotheses and taxon pulses (Galbreath & Hoberg, 2012). *Proteocephalus fallax* population differentiation between the subarctic and perialpine regions supports the two-refugia origin of European whitefish recolonization of Western Europe (Østbye et al., 2005), the N clade west of the Ural Mountains, and the C clade from near the mouth of the Rhine. *Proteocephalus fallax* high degree of divergence between Langfjordvatn (Pasvik River system, but isolated from it for ~9,000 YBP), and Suohpatjávri (Alta River catchment), together with high inbreeding values and completely segregated cytochrome c oxidase subunit 1 haplotypes (Brabec et al., 2023) indicate a low number of founders and strong genetic drift as the main drivers of allopatric differentiation. Thus, this recent taxon pulse resulted in the vicariance of both, the tapeworm and the European whitefish (Østbye et al., 2006; Praebel et al., 2013), during the early postglacial recolonization of these lakes. In the Swiss Alps, European whitefish cooccurrence from two glacial refugia led to a hybridogenic ancestral population that underwent rapid adaptive radiations, in at least 5 lake/super-lake systems independently (De-Kayne et al., 2022; Hudson et al., 2011), with three of the major European whitefish genetic clusters being included in our study. Whereas *Coregonus* spp. from the perialpine lakes Thun-Brienz and Walen are more closely related to each other than either of them to populations from lake Bienne in midland (De-Kayne et al., 2022), the higher divergence of *P*. *fallax* detected between Walen and Thun-Brienz speaks to multiple recolonization waves of the perialpine lakes [similar to, e.g., sculpin fish Lucek et al. (2018)]. Though, the influence of historical human-made changes to the connectivity and introductions of whitefish fry (Selz et al., 2020) cannot be excluded. *Proteocephalus fallax* strong differentiation between the perialpine and the subarctic lakes suggests that the northern European whitefish lineage that recolonized the Alps arrived uninfected, or that the ancestral tapeworm associated with the northern European whitefish lineage was replaced by a different *P*. *fallax* lineage that was acquired along the colonization path at intermediate location(s). The loss of parasites, or their lagged arrival, favors reduced parasite pressure (low parasite prevalence) when hosts expand into new regions (Phillips et al., 2010), and predicts an immediate “ecological release” favoring elevated host population growth rates and persistence at the expansion front, followed by an “evolutionary release” (Alexander et al., 2022) for both the host and the parasite. In the context of adaptive radiations through ecological speciation, different mechanisms may contribute to the parasite-driven mediated selection of the host (Karvonen & Seehausen, 2012), but evidence is still limited (Karvonen et al., 2013). We propose that the escape from parasites with the initial “honeymoon phase” *sensu*Phillips et al. (2010) and “evolutionary release” *sensu*Alexander et al. (2022) contributed to the initial burst of postglacial whitefish diversification, with a delayed arrival, and simultaneous opportunistic tapeworm host repertoire expansion into emerging *Coregonus* species flocks. Although today *P*. *fallax* may only cause a mild fitness loss to the host by removing nutrients (but notice the high infection levels reported in Results), in a situation of high competition for limited resources in recently formed oligotrophic lakes, escaping infection could confer a considerable advantage. Furthermore, delays in dynamics of colonization and taxon pulses have been observed in other complex host–parasite systems and predicted by models in the framework of the SP (Araujo et al., 2015; Galbreath & Hoberg, 2015; Galbreath et al., 2023; Haas et al., 2020).

### Host adaptive radiation results in parasite host–repertoire expansion and subsequent differentiation

Parasite diversification in the context of the adaptive radiation cascade has been sparsely investigated hitherto, with most studies focused on the radiation of cichlids of West African lakes and their direct life cycle monogenean parasites (e.g., Mendlová et al., 2012; Vanhove et al., 2015) that show codifferentiation and predominantly high host specificity. Conversely, we found that gene flow has constrained the differentiation of *P*. *fallax* subpopulations among sympatric European whitefish hosts relative to that of its fish hosts during the recent postglacial expansion and within-lake radiations. Even more, the intralake differentiation of this tapeworm was unrelated to the genetic differentiation among sympatric European whitefish hosts, which rules out codivergence with the host (Althoff et al., 2014) and supports the view that codiversification simultaneously with the host is not a common mode of parasite speciation (Agosta & Brooks, 2020; de Vienne et al., 2013). Macroevolutionary studies of typically highly host-specific parasites attribute the lack of codifferentiation to parasite life history traits like having either additional hosts in the life cycle as in the case of *P*. *fallax*, stages with short periods of transmission in the environment, possibly facilitating (definitive) host switching (Huyse & Volckaert, 2005; Paterson & Poulin, 1999), or the host ecology and social behavior (Desdevises et al., 2002; Kmentová et al., 2020) among others (Larose & Schwander, 2016). These are all factors that increase the opportunities for encountering alternative hosts. In the context of the SP (Agosta & Brooks, 2020; Nylin et al., 2018), *Proteocephalus fallax* opportunity and capacity to extend the host range in the European whitefish radiation is facilitated by ecological fitting to whitefish resources via phylogenetic conservatism (Agosta & Klemens, 2008; Brooks & McLennan, 2012) among species flocks at different stages of diversification along the speciation continuum.

By integrating ecological factors that can shape parasite microevolution and underpin macroevolutionary patterns (Blasco-Costa et al., 2021), we revealed that phenotypic diversification in European whitefish exploitation of new ecological niches fosters an incipient nonadaptive radiation cascade in *P*. *fallax*. Our results support the oscillation hypothesis (Brooks et al., 2019; Janz & Nylin, 2008), alternating between *P*. *fallax* expansion into available European whitefish resources followed by an exploitation (specialization) phase, which progresses with a time-lag as predicted by models (Araujo et al., 2015; Braga et al., 2018). Differentiation along the same water-depth ecological axis of lakes as its *Coregonus* hosts is most likely associated with the use of different intermediate crustacean hosts for transmission. Three lines of evidence led us to propose this hypothesis. First, parasite intra-lacustrine population substructure, though weak, across European whitefish species/ecomorphs and both, their trophic preference and habitat use, supports a scenario of recent parasite differentiation with gene flow conditional on ecological opportunity (see Hoberg & Brooks, 2008). Second, variation in parasite population genetic parameters correlated with lake depth and oxygenation, both environmental characteristics important for whitefish speciation (typically occurring along depth clines) to the point that changes to the availability of pelagic and profundal habitats have caused speciation reversal (Bhat et al., 2014; Vonlanthen et al., 2012). Third, larval stages of *P*. *longicolliss.l.* are known to infect several crustacean taxa in Europe (Scholz, 1999 and references within). Given that salmonid trophic niche variation depends on prey diversity (Sánchez-Hernández et al., 2021), and that European whitefish adaptive radiation was driven by colonization of new habitats (e.g., littoral, pelagic, and profundal) and trophic specialization in postglacial lakes (e.g., Ingram et al., 2012; Siwertsson et al., 2013), it is likely that the differential use of planktonic and benthic crustaceans via ecological fitting in sloppy fitness space (Agosta & Klemens, 2008; Agosta et al., 2010) promotes *P*. *fallax* population substructure detected across sympatric whitefish hosts. Thus, *P*. *fallax* may represent another example of “multiple-level ecological fitting” (Malcicka et al., 2015) by tracking both fish and intermediate host available resources in the postglacial lakes. These results also highlight the need to stop overlooking the identity and diversity of intermediate hosts if we seek understanding the mechanisms of parasite evolution (Blasco-Costa & Poulin, 2017).

### Linking micro- and macroevolutionary research

Differentiation of a parasite is often associated with its most mobile host (Blasco-Costa & Poulin, 2013; Louhi et al., 2010; Prugnolle et al., 2005), but see Mazé-Guilmo et al. (2016). Our findings highlight that dispersal with the fish host during taxon pulses, and founder events associated with the recolonization and establishment of allopatric populations, predispose to high fixation rates (i.e., increased divergence rates) in parasite populations and foster spatial differentiation patterns at micro-, and possibly vicariance later at macroevolutionary time scales. Allopatry predisposed lice to codivergence with its hosts compared to when hosts were found in contact zones (Barker, 1994). In a microsporidian parasite and its aquatic host, shared biogeographic histories and dispersal explained the congruent diversification of both organisms at the regional scale (Park et al., 2020). Taken all together, we propose that host-dependent parasite dispersal during taxon pulses, coupled with parasite founder events during colonizations in range expansions represent important ecological and evolutionary processes generating the pattern of parasite phylogenetic tracking of their hosts in space. In other words, these processes alone could explain some mimicked patterns of codiversification/codifferentiation (correlated diversification between interacting lineages (Clayton et al., 2015)). Microevolutionary research across geographical scales will further reveal processes and underlying mechanisms behind parasite differentiation and eco-evolutionary change.

## Supplementary material

Supplementary material is available online at *Evolution Letters*.

qrae025_suppl_Supplementary_Material

qrae025_suppl_Supplementary_Table_S2

qrae025_suppl_Supplementary_Table_S3

## Data Availability

All collapsed and paired-end sequence data for samples sequenced in this study are available in compressed fastq format through NCBI’s BioProject no. PRJNA910576 (ddRAD accessions SAMN32132446–959, reference genome accession SAMN32134074), together with rescaled and trimmed bam sequence alignments against both the nuclear and mitochondrial reference genome. All scripts used to perform the analyses presented in this paper are available through https://github.com/JeremyLGauthier/Scripts_Proteocephalus.
