# Supplementary material for: Testing the radiation cascade in postglacial radiations of whitefish and their parasites: founder events and host ecology drive parasite evolution
Source: Evol Lett. 2024 Jun 19;8(5):706–18. doi: 10.1093/evlett/qrae025 (PMC11424076; doi:10.1093/evlett/qrae025)
Supplement: qrae025_suppl_Supplementary_Material [file qrae025_suppl_supplementary_material.docx]

**Supplementary Methods**

**Fish sampling, identification and infection statistics**

European whitefish populations were target-sampled between July and December 2017. Most fish from the perialpine lakes Bienne, Brienz and Thun were obtained from commercial local fishery catches from mid-July 2017 to mid-January 2018, while some fish from the lake Bienne and most fish from lake Walen were obtained in December 2017 on spawning grounds known to local fishermen. Subarctic lakes Langfjordvatn and Suohpatjávri were sampled with benthic and pelagic gillnets to target the three principal habitats, i.e. littoral, profundal, and pelagic, respectively, during September-October 2017. Fin clips were preserved in ethanol for genotyping, and identification to the species/ecotype level was done based on the results of the STRUCTURE clustering analyses (Pritchard *et al.* 2000) and morphology (see below).

*Proteocephalus fallax* worms were extracted from the fish intestine, individually rinsed in saline, and immediately preserved in separate vials in absolute ethanol. The prevalence of infection (percentage of whitefish infected with *P*. *fallax* amongst all fish examined), and the mean intensity of infection (number of *P*. *fallax* specimens in infected fish) were calculated for each lake, and whitefish species within lake as measures of population census size**.**

**Genotyping of European whitefish specimens and species/ecotypes assignments**

European whitefish DNA was extracted from fin clips with a standard protocol using 5% Chelex solution and Proteinase K.

Perialpine *Coregonus* spp. individuals were genotyped using 10 microsatellite loci of Patton et al. (1997), Turgeon et al. (1999) and Rogers et al. (2004) organized in two multi-plex PCRs (see table below for details on the organization of the microsatellite loci into multiplexes for Norwegian (left) and Swiss European whitefish (right), and the final concentrations of individual primers in PCR assays).

| **Norwegian microsatellite loci** | | | **Swiss microsatellite loci** | | |
| --- | --- | --- | --- | --- | --- |
| **Locus name** | **Mplx** | **Conc (μM)** | **Locus name** | **Mplx** | **Conc (μM)** |
| Bwf1 | 1 | 0.25 | Cocl-Lav06 | 1 | 0.04 |
| Bwf2 | 1 | 0.1 | Cocl-Lav45 | 1 | 0.04 |
| CalTet-18 | 1 | 0.2 | Cocl-Lav49 | 1 | 0.04 |
| Cocl-Lav04 | 1 | 0.08 | Cocl-Lav61 | 1 | 0.04 |
| Cocl-Lav06 | 1 | 0.15 | Cocl-Lav68 | 1 | 0.04 |
| Cocl-Lav27 | 1 | 0.04 | C2-157 | 1 | 0.04 |
| CalTet-03 | 1 | 0.125 | Bwf2 | 2 | 0.08 |
| CalTet-13 | 1 | 0.15 | Cocl-Lav04 | 2 | 0.05 |
| Cocl-Lav10 | 1 | 0.05 | Cocl-Lav10 | 2 | 0.04 |
| Cocl-Lav52 | 2 | 0.25 | Cocl-Lav18 | 2 | 0.04 |
| Cocl-Lav49 | 2 | 0.08 |  |  |  |
| BFRO018 | 2 | 0.08 |  |  |  |
| CalTet-10 | 2 | 0.12 |  |  |  |
| Cocl-Lav18 | 2 | 0.08 |  |  |  |
| CalTet-05 | 3 | 0.2 |  |  |  |
| CalTet-09 | 3 | 0.1 |  |  |  |
| CalTet-15 | 3 | 0.1 |  |  |  |
| CalTet-06 | 3 | 0.15 |  |  |  |
| C2-157 | 4 | 0.1 |  |  |  |
| CalTet-01 | 4 | 0.15 |  |  |  |
| CalTet-12 | 4 | 0.1 |  |  |  |
| CalTet-17 | 4 | 0.15 |  |  |  |

Both PCRs were run as 5μl reactions, using 2.5 μl of the 2× Multiplex PCR Master Mix (Qiagen), 0.25 μl of the primer mix, 0.6 μl of template DNA and 1.65 μl of water. The cycling conditions were identical for both PCRs: an initial denaturation of 15 min at 95 °C, followed by 32 cycles of 15 s at 95 °C, 90 s at 57 °C, 90 s at 72 °C and a final elongation 30 min at 60 °C. Fragment analyses were run by a commercial service provider SeqMe (Czech Republic) using GeneScan™ 600 LIZ® internal size standard on the ABI3130xl genetic analyzer. Allele sizes were determined and manually checked with the Microsatellite plugin of Geneious Prime (http://www.geneious.com) using the same scoring panel as Doenz et al. (2018). Supplementary Table S3 lists the bin values of these loci.

Subarctic European whitefish were genotyped with a set of 22 microsatellite loci organized in four multiplex sets (see table above) following the methodology of previous studies on the European whitefish in Fennoscandia by Præbel et al. (2013). All four PCRs were run as 2.5μl reactions, using 1.25 μl 2× Multiplex PCR Master Mix, 0.25 μl of the primer mix, 0.5 μl of template DNA and 0.5 μl of water. The cycling conditions for all multiplex assays consisted of an initial denaturation 15 min at 95 °C and a ﬁnal elongation 30 min at 60 °C. The ampliﬁcation proceeded through: i) 25 cycles of 30 s at 95 °C 3 min at 57 °C, and 1 min at 72 °C; ii) 25 cycles of 30 s at 95 °C, 3 min at 60 °C, and 1 min at 72 °C; iii) 26 cycles of 30 s at 95 °C, 3 min at 61 °C, and 1 min at 72 °C; iv) 27 cycles of 30 s at 95 °C, 3 min at 60 °C, and 1 min at 72 °C. Fragment analyses were run on an identical instrument as in Häkli et al. (2018) using GeneScan™ 500 LIZ® internal size standard on the ABI3130xl genetic analyzer. Allele sizes were determined and manually checked with the Microsatellite plugin of Geneious Prime using the binsets of Häkli et al. (2018). The verification resulted in exclusion of five microsatellite loci (Bwf1, Bwf2, C2-157, CalTet-10, CalTet-5) due to a high proportion of unreadable peaks across specimens as well as ambiguous bin assignments. Supplementary Table S3 lists the bin values of the remaining 17 loci.

**Hierarchical cluster analysis**

All the STRUCTURE analyses were run using the StrAuto script (Chhatre & Emerson, 2017) running 10 replicates of 1,000,000 iterations (100,000 burn-in steps) using the admixture LOCPRIOR model and correlated allele frequency. Structure Harvester (Earl & vonHoldt, 2012) and CLUMPP (Jakobsson & Rosenberg 2007) were used to analyze STRUCTURE results and generate consensual assignment proportions from the 10 structure runs, respectively.

Hierarchical cluster analyses were run for each lake separately, and each initial STRUCTURE analysis included all newly genotyped fish from a given lake (i.e., not just those infected with *P. fallax*). In the case of perialpine lakes Bienne, Brienz, Thun and Walen, newly genotyped European whitefish individuals were combined with the lake representatives characterized in Doenz et al. (2018) and Selz et al. (2020). After each run, the dataset was divided into two subsets according to the K=2 assignments (cut-off value set at 50) and submitted to another hierarchical STRUCTURE run until all subsets supported the K value of 1. Resulting genetic clusters (Figure 1 below) were then assigned to known European whitefish species according to the reference panel whitefish individuals of Doenz et al. (2018) that were included in the dataset. Since the hierarchical cluster analysis had only enough power to differentiate 3 and 5 genetic clusters in Brienz and Thun, respectively, further assignment into the 4 and 6 European whitefish species present in those lakes was based on the fish morphological data collected along the genetic data.

** Figure 1**. Individual steps of the hierarchical clusters analyses of *Coregonus* spp./ecotypes for each lake.

**Parasite whole genome sequencing and assembly**

We selected six large adult *P*. *fallax* specimens of about 4 cm in length, with maximum body width of 1 mm at the posterior mature proglottid. After removal from the host, the specimens were rinsed and brushed in physiological solution while alive, and directly subjected to a tissue lysis. Each time, gDNA was extracted from a single *P*. *fallax* specimen using MagAttract^Ⓡ^ HMW DNA kit (Qiagen) and eluted in 100 µL AE Buffer. Genomic DNA quantities and qualities were checked using Qubit fluorometer (Invitrogen) and Fragment Analyzer (Agilent), respectively, resulting in gDNA concentrations ranging from 35 to 73 ng/µL, and mean fragment size from 6 to 36 Mb across samples. The extraction with the highest concentration and longest fragments obtained from a single *P*. *fallax* specimen of a whitefish from Lake Geneva (Switzerland) was selected for sequencing. 400 ng of HMW gDNA was used for Oxford Nanopore long-read library preparation using the Rapid Sequencing Kit. Two libraries of the same sample were sequenced sequentially (after washing the flow cell with the Flow Cell Wash Kit) on a FLO-MIN106 flow cell for a total of 46 hours using MinION 1B sequencer (Oxford Nanopore^TM^ Technologies). Base calling was performed with GUPPY software using the High-Accuracy models. An Illumina TruSeq DNA PCR-free library with insert sizes of 550 bp was constructed from the same gDNA extract, according to the Illumina standard protocol and subjected to 150 bp paired-end sequencing on a NovaSeq 6000 platform (Illumina). Read quality was assessed using FastQC (Andrews 2010). Genome size estimation was carried out on Illumina reads using a k-mer depth frequency distribution analysis performed with KMC3 (Kokot et al., 2017) and GenomeScope2.0 (Ranallo-Benavidez et al., 2020).

**Performance evaluation of restriction enzymes for the ddRAD of *Proteocephalus fallax***

Restriction digestion tests were run as 9μL reactions using 1.7 μL of water, 0.9 μL of 10× CutSmart buffer, 0.2 μL (2 U) of each restriction enzyme (New England Biolabs; see titles of Fragment Analyzer profiles in Figure 2 below for enzyme combinations), and 6 μL of template DNA (7 ng/μL). Reactions were incubated at 37 °C for 3 hours on a thermal cycler with a heated lid and kept at 4 °C afterwards. Purifications with SPRI beads (CleanNGS, CleanNA) with the beads to sample ratio 2:1 were performed according to the manufacturer's instructions and eluted with a 9.5 μL EB buffer (9 μL retrieved).

**Figure 2**. Restriction fragment profiles with different combinations of restriction enzymes.

**Detailed protocol for ddRAD sequencing of parasitic Platyhelminthes**

This protocol is based on the original ddRAD protocol of Peterson *et al*. (2012) with modifications from Mastretta-Yanes *et al.* (2015) and customized to the specifics of the parasitic flatworm genomes. It includes the following most important modifications:

– Combining NlaIII and MseI restriction endonucleases to perform gDNA digestion.

– Carrying out the size selection after the PCR amplification on equimolarly pooled samples tagged with single index.

– Use of CleanNGS (CleanNA) magnetic beads to perform DNA purification steps throughout the protocol.

– Visualization of DNA size profiles on the Fragment Analyzer (Agilent).

– Size selection of fragments of 320-500 bp on Blue Pippin (Sage Science).

**Step 0.** PREPARE STARTING REAGENTS

Genomic DNA

The target amount of 60 ng gDNA per each specimen (minimum sample concentration of 10 ng/μL) was used as the starting gDNA amount for double digestion.

Adapter sequences

P1.1_NlaIII ACACTCTTTCCCTACACGACGCTCTTCCGATCTxxxxxxCATG

P1.2_NlaIII xxxxxxAGATCGGAAGAGCGTCGTGTAGGGAAAGAGTGT

Note: The string of Xs in P1.1 represents the 6nt unique barcode, Xs in P1.2 is the reverse complement of the barcode.

P2.1_MseI GTGACTGGAGTTCAGACGTGTGCTCTTCCGATCT

P2.2_MseI TAAGATCGGAAGAGCGAGAACAA

Illumina PCR oligo sequences

IILPCR1short AATGATACGGCGACCACCGAGATCTACACTCTTTCCCTACACGACG

Note: The ILLPCR1short oligo anneals to the P1_NlaIII adapter sequence

ILLPCR2 CAAGCAGAAGACGGCATACGAGATxxxxxxGTGACTGGAGTTCAGACGTGTGC

Note: The ILLPCR2 oligo anneals to the P2.1_MseI adapter sequence. The string of Xs in this oligo represents the 6nt Illumina index.

Adapter annealing

Adapter P1: Combine 0.1 μL of P1.1_NlaIII (100 μM) and 0.1 μL of P1.1_NlaIII (100 μM) oligos with 0.8 μL of Annealing buffer (0.1M Tris-HCl, 0.5M NaCl) to get 1μM of each oligo.

Note: Prepare enough for all specimens carrying the same barcode. Include 1.8x pipetting error.

Adapter P2: Combine 0.1 μL of P2.1_MseI (100 μM) and 0.1 μL of P2.2_MseI (100 μM) oligos with 0.8 μL of Annealing buffer (0.1M Tris-HCl, 0.5M NaCl) to get 1μM of each oligo.

Note: Prepare enough for all specimens carrying the same barcode. Include 1.8x pipetting error.

Anneal oligos in a thermal cycler. Program: 95 °C /1 min, followed by a gradual decrease of 0.1 °C/1 sec to 20 °C. Store aliquots of annealed adapters in a freezer.

Note: Keep adapters organized in strips for compatible use later with a multichannel pipette to set reactions.

**Step 1.** RESTRICTION ENZYME DOUBLE DIGESTION

**1a** Prepare Master Mix for N reactions (include 1.02x pipetting error)

| reagent | Producer/N° Article | volume [μL] for 1 sample |
| --- | --- | --- |
| H2O | Millipore/H20MB0506 | 1,9 |
| 10x CutSmart buffer | Provided with enzymes | 0,9 |
| MseI (10,000 U/mL) | NEB/R0525S | 0,1 |
| NlaIII (10 000 U/mL) | NEB/R0125S | 0,1 |
| Vtot Mix |  | 3 |

Note: Distribute Master Mix 1 in strips.

**1b** Mix with 6 μL of DNA (~10 ng/μL)

**1c** Briefly spin the strips

**1d** Incubate at 37 °C for 3 hours in a thermal cycler with a heated lid. Hold at 4 °C.

**1e** Perform purification with CleanNGS SPRI beads:sample ratio 2:1 according to the general protocol:

*General CleanNGS magnetic beads clean-up protocol

***a** Bring CleanNGS magnetic beads bottle to room temperature (30 min or more)

***b** Prepare fresh 70% EtOH by mixing e.g. 35 mL of 100% EtOH and 15 mL of H_2_O measured separately. Never top-up EtOh with H2O as it results in more diluted ethanol.

***c** Add the correct amount of SPRI beads according to the volume specified in the protocol (e.g. 18 μL of SPRI when mixing with 9 μL of sample if 2:1 sample:bead ratio is prescribed) and mix by pipetting up and down 20+ times. Let stand for 5 mins. Place on a magnetic stand and let bind for 5 mins.

***d** Without moving the strip from the magnetic stand, aspirate the cleared solution & discard.

***e** Add 180 μL of 70% EtOH with a pipette and let stand at RT for 1 min.

***f** Without moving the strip from the magnetic stand, aspirate the cleared solution & discard.

***g** Add 180 μL of 70% EtOH for the second wash and let stand at RT for 1 min.

***h** Without moving the strip from the magnetic stand, aspirate the cleared solution & discard. Check for any residual drops of ethanol. Remove with a small pipette tip if necessary.

***i** Let the beads dry briefly (in laminar flow, this takes only a few seconds). Do not overdry the beads!

***j** Remove the strip from the magnetic stand. Add the correct amount of EB buffer or water, according to the volume specified in the protocol. Mix by pipetting up and down 10+ times or until no beads stick to the strip tube wall. Let stand for 5 mins.

***k** Place the strip on the magnetic stand. Let stand for 5 mins.

***l** Take 0.5 μL less than the total elution volume used and transfer to a new strip.

**1f** Elute with 9 μL of EB Buffer.

**Step 2.** ADAPTER LIGATION (includes barcoding)

**2a** Prepare Master Mix for N reactions (include 1.2x pipetting error)

| reagent | Producer/N° Article | volume [μL] for 1 sample |
| --- | --- | --- |
| Pink H2O | Millipore/H20MB0506 | 0,0565 |
| 10x CutSmart buffer | Provided with enzymes | 0,26 |
| T4 DNA ligase (400 U/μl) | NEB/M0202S, M0202L | 0,1675 |
| 100 mM ATP | ThermoFisher/R0441 | 0,116 |
| RAD MseI adaptor (10 μM) | See "OLIGOS" | 1 |
| RAD NlaIII adaptor (10 μM) | See "OLIGOS" | 1 |
| Vtot [ul] |  | 2,6 |

Note: Various NlaIII adapters might be used in separate reaction mixes and thus, NlaIII adapters might need to be added individually in later steps.

**2b** Mix 2.6 μL of the mixture with the 9 μL of the double-digested gDNA from Step 1.

**2c** Briefly spin the strips

**2d** Incubate at 16 °C for 3 hours in a thermal cycler with a heated lid (40 °C). Hold at 4 °C.

**2e** Dilute with 38.6 μL H_2_O to bring to a total volume of 50 μL.

**2f** Perform purification with CleanNGS SPRI beads:sample ratio 1.2:1.

**2g** Elute with 20 μL of EB Buffer.

**Step 3.** PCR AMPLIFICATION (includes indexing)

**3a** Prepare separate Master Mixes (include 1.2x pipetting error) for individual Illumina primers carrying separate indexes. To compensate for stochastic differences during PCR amplification of a huge mixture of fragments, run three PCR replicates per each Master mix.

| Reagent | Producer/N° Article | volume [μL] for 3 PCR |
| --- | --- | --- |
| Pink H2O | Millipore/H20MB0506 | 0,45 |
| 5X SuperFi™ Buffer | Thermofisher | 6 |
| dNTP mix (25 mM each) | Thermofisher/R0182 | 0,24 |
| Platinum SuperFi™ Polym (2U/uL) | Thermofisher/12351010 | 0,3 |
| 5X SuperFi™ GC Enhancer | Thermofisher | 6 |
| VTOT |  | 12,99 |
|  | | |
| PCR primer Mix (5 μM each) | See "OLIGOS" | 2,01 |

Note: Prepare the Master Mix without the PCR primer mix. Divide the total volume by the number of indexes to be used. Add the individual PCR primer mix to the Master Mix.

**3b** Distribute the Master Mix in a plate by 15 μL.

**3c** Mix with 15 μL of the purified ddDNA with ligated adapters from Step 2.

**3d** Distribute 10 μL in another 2 plates to create a total of 3 replicates of each PCR.

**3e** Place in a thermal cycler with a heated lid and run the following protocol:

| 98 ℃ | 30 sec |  |
| --- | --- | --- |
| 98 ℃ | 20 sec | 20 cycles |
| 60 ℃ | 30 sec |  |
| 72 ℃ | 40 sec |  |
| 72 ℃ | 2 min |  |

**3f** Pool the 3 replicates in 1 plate.

**3g** Prepare the following Master Mix

| Reagent | Producer/N° Article | Volume [μL] for 3 pooled replicates |
| --- | --- | --- |
| Pink H2O | Millipore/H20MB0506 | 0,15 |
| 5X SuperFi™ Buffer | Thermofisher | 0,6 |
| dNTP mix (25 mM each) | Thermofisher/R0182 | 0,24 |
| PCR primer Mix (5 μM each) | See "OLIGOS" | 2,01 |
| VTOT |  | 3 |

**3h** Add 3 μL of the Master mix to each well with the pooled PCR product.

**3i** Place in a thermal cycler with a heated lid and run the following protocol:

| 98 ℃ | 3 min |
| --- | --- |
| 60 ℃ | 2 min |
| 72 ℃ | 12 min |

**3j** Perform purification with CleanNGS SPRI beads:sample ratio 0.8:1.

**3k** Elute with 20 μL of Millipore H_2_O.

Note: Use Millipore H_2_O here instead of the buffer to allow for subsequent concentration of libraries without increasing the relative salt concentration.

**Step 4.** LIBRARY QUANTIFICATION & POOLING

***** Perform quality check by running several randomly selected samples on a Fragment Analyzer to check if the library profiles are similar and that equimolar pools can be prepared based on the individual library concentrations.

**4a** Quantify individual libraries with a fluorescent dye-based assay, e.g. Qubit assays (Invitrogen).

**4b** Prepare equimolar pools of all of the libraries carrying the same Illumina index.

**4c** Concentrate individual pools (index-specific) with a SpeedVac to a target volume of 30 μL.

**4d** Re-quantify the pooled libraries with a fluorescent dye-based assay.

**Step 5.** SIZE SELECTION

**5a** Use Blue Pippin (Sage Science) 2% agarose gel cassettes (with internal marker) to separate fragments of desired size.

Note: The maximum volume to load on a cassette is 30 μL, the maximum DNA quantity is 10 μg. We used a broad-range setting, selecting fragments of 320-500 bp, using 2% DF Marker V1 cassettes.

**5b** Quantify the size-selected libraries again using a fluorescent dye-based assay.

**5c** Check the profiles of the size-selected libraries on a Fragment Analyzer to confirm that the desired size selection was achieved.

**Step 6.** LIBRARY SEQUENCING

6a Pool all libraries in an equimolar proportion into a single 1.5 mL eppendorf tube.

6b Dispatch the final pool to a sequencing facility for Illumina sequencing.

Note: We sequenced our samples on 6 lanes of Illumina HiSeq 2500 and 150bp paired-end reads.

**Barcoded adapter sequences used in this study**

| NlaIII_P1.1_011 | ACACTCTTTCCCTACACGACGCTCTTCCGATCTAAGACCCATG |
| --- | --- |
| NlaIII_P1.1_061 | ACACTCTTTCCCTACACGACGCTCTTCCGATCTTTCGTTCATG |
| NlaIII_P1.1_071 | ACACTCTTTCCCTACACGACGCTCTTCCGATCTATGAATCATG |
| NlaIII_P1.1_081 | ACACTCTTTCCCTACACGACGCTCTTCCGATCTGGTCGGCATG |
| NlaIII_P1.1_091 | ACACTCTTTCCCTACACGACGCTCTTCCGATCTGACGATCATG |
| NlaIII_P1.1_241 | ACACTCTTTCCCTACACGACGCTCTTCCGATCTTCTGGTCATG |
| NlaIII_P1.1_261 | ACACTCTTTCCCTACACGACGCTCTTCCGATCTCAGCAGCATG |
| NlaIII_P1.1_271 | ACACTCTTTCCCTACACGACGCTCTTCCGATCTCCGCTCCATG |
| NlaIII_P1.1_281 | ACACTCTTTCCCTACACGACGCTCTTCCGATCTGTATGCCATG |
| NlaIII_P1.1_351 | ACACTCTTTCCCTACACGACGCTCTTCCGATCTAGGCTACATG |
| NlaIII_P1.1_371 | ACACTCTTTCCCTACACGACGCTCTTCCGATCTCATTCACATG |
| NlaIII_P1.1_451 | ACACTCTTTCCCTACACGACGCTCTTCCGATCTTGACCGCATG |
| NlaIII_P1.1_461 | ACACTCTTTCCCTACACGACGCTCTTCCGATCTCTAACGCATG |
| NlaIII_P1.1_471 | ACACTCTTTCCCTACACGACGCTCTTCCGATCTTATTAGCATG |
| NlaIII_P1.1_481 | ACACTCTTTCCCTACACGACGCTCTTCCGATCTGTCTAGCATG |
| NlaIII_P1.1_501 | ACACTCTTTCCCTACACGACGCTCTTCCGATCTTGCTTCCATG |
| NlaIII_P1.1_531 | ACACTCTTTCCCTACACGACGCTCTTCCGATCTAATGGCCATG |
| NlaIII_P1.1_541 | ACACTCTTTCCCTACACGACGCTCTTCCGATCTTTGCGCCATG |
| NlaIII_P1.1_591 | ACACTCTTTCCCTACACGACGCTCTTCCGATCTGAGAGTCATG |
| NlaIII_P1.1_611 | ACACTCTTTCCCTACACGACGCTCTTCCGATCTCGAATACATG |
| NlaIII_P1.1_631 | ACACTCTTTCCCTACACGACGCTCTTCCGATCTGCAGGACATG |
| NlaIII_P1.1_661 | ACACTCTTTCCCTACACGACGCTCTTCCGATCTAGAGCACATG |
| NlaIII_P1.1_691 | ACACTCTTTCCCTACACGACGCTCTTCCGATCTACCTAACATG |
| NlaIII_P1.1_721 | ACACTCTTTCCCTACACGACGCTCTTCCGATCTCCTACTCATG |
| NlaIII_P1.2_011 | GGTCTTAGATCGGAAGAGCGTCGTGTAGGGAAAGAGTGT |
| NlaIII_P1.2_061 | AACGAAAGATCGGAAGAGCGTCGTGTAGGGAAAGAGTGT |
| NlaIII_P1.2_071 | ATTCATAGATCGGAAGAGCGTCGTGTAGGGAAAGAGTGT |
| NlaIII_P1.2_081 | CCGACCAGATCGGAAGAGCGTCGTGTAGGGAAAGAGTGT |
| NlaIII_P1.2_091 | ATCGTCAGATCGGAAGAGCGTCGTGTAGGGAAAGAGTGT |
| NlaIII_P1.2_241 | ACCAGAAGATCGGAAGAGCGTCGTGTAGGGAAAGAGTGT |
| NlaIII_P1.2_261 | CTGCTGAGATCGGAAGAGCGTCGTGTAGGGAAAGAGTGT |
| NlaIII_P1.2_271 | GAGCGGAGATCGGAAGAGCGTCGTGTAGGGAAAGAGTGT |
| NlaIII_P1.2_281 | GCATACAGATCGGAAGAGCGTCGTGTAGGGAAAGAGTGT |
| NlaIII_P1.2_351 | TAGCCTAGATCGGAAGAGCGTCGTGTAGGGAAAGAGTGT |
| NlaIII_P1.2_371 | TGAATGAGATCGGAAGAGCGTCGTGTAGGGAAAGAGTGT |
| NlaIII_P1.2_451 | CGGTCAAGATCGGAAGAGCGTCGTGTAGGGAAAGAGTGT |
| NlaIII_P1.2_461 | CGTTAGAGATCGGAAGAGCGTCGTGTAGGGAAAGAGTGT |
| NlaIII_P1.2_471 | CTAATAAGATCGGAAGAGCGTCGTGTAGGGAAAGAGTGT |
| NlaIII_P1.2_481 | CTAGACAGATCGGAAGAGCGTCGTGTAGGGAAAGAGTGT |
| NlaIII_P1.2_501 | GAAGCAAGATCGGAAGAGCGTCGTGTAGGGAAAGAGTGT |
| NlaIII_P1.2_531 | GCCATTAGATCGGAAGAGCGTCGTGTAGGGAAAGAGTGT |
| NlaIII_P1.2_541 | GCGCAAAGATCGGAAGAGCGTCGTGTAGGGAAAGAGTGT |
| NlaIII_P1.2_591 | ACTCTCAGATCGGAAGAGCGTCGTGTAGGGAAAGAGTGT |
| NlaIII_P1.2_611 | TATTCGAGATCGGAAGAGCGTCGTGTAGGGAAAGAGTGT |
| NlaIII_P1.2_631 | TCCTGCAGATCGGAAGAGCGTCGTGTAGGGAAAGAGTGT |
| NlaIII_P1.2_661 | TGCTCTAGATCGGAAGAGCGTCGTGTAGGGAAAGAGTGT |
| NlaIII_P1.2_691 | TTAGGTAGATCGGAAGAGCGTCGTGTAGGGAAAGAGTGT |
| NlaIII_P1.2_721 | AGTAGGAGATCGGAAGAGCGTCGTGTAGGGAAAGAGTGT |

**Illumina indexed oligonucleotide sequences used in this study**

| ILLPCR2_01 | CAAGCAGAAGACGGCATACGAGATcgtgatGTGACTGGAGTTCAGACGTGTGC |
| --- | --- |
| ILLPCR2_04 | CAAGCAGAAGACGGCATACGAGATtggtcaGTGACTGGAGTTCAGACGTGTGC |
| ILLPCR2_05 | CAAGCAGAAGACGGCATACGAGATcactgtGTGACTGGAGTTCAGACGTGTGC |
| ILLPCR2_06 | CAAGCAGAAGACGGCATACGAGATattggcGTGACTGGAGTTCAGACGTGTGC |
| ILLPCR2_08 | CAAGCAGAAGACGGCATACGAGATtcaagtGTGACTGGAGTTCAGACGTGTGC |
| ILLPCR2_11 | CAAGCAGAAGACGGCATACGAGATgtagccGTGACTGGAGTTCAGACGTGTGC |
| ILLPCR2_12 | CAAGCAGAAGACGGCATACGAGATtacaagGTGACTGGAGTTCAGACGTGTGC |
| ILLPCR2_13 | CAAGCAGAAGACGGCATACGAGATttgactGTGACTGGAGTTCAGACGTGTGC |
| ILLPCR2_14 | CAAGCAGAAGACGGCATACGAGATggaactGTGACTGGAGTTCAGACGTGTGC |
| ILLPCR2_15 | CAAGCAGAAGACGGCATACGAGATtgacatGTGACTGGAGTTCAGACGTGTGC |
| ILLPCR2_16 | CAAGCAGAAGACGGCATACGAGATggacggGTGACTGGAGTTCAGACGTGTGC |
| ILLPCR2_17 | CAAGCAGAAGACGGCATACGAGATctctacGTGACTGGAGTTCAGACGTGTGC |
| ILLPCR2_18 | CAAGCAGAAGACGGCATACGAGATgcggacGTGACTGGAGTTCAGACGTGTGC |
| ILLPCR2_22 | CAAGCAGAAGACGGCATACGAGATcgtacgGTGACTGGAGTTCAGACGTGTGC |
| ILLPCR2_23 | CAAGCAGAAGACGGCATACGAGATccactcGTGACTGGAGTTCAGACGTGTGC |
| ILLPCR2_24 | CAAGCAGAAGACGGCATACGAGATgctaccGTGACTGGAGTTCAGACGTGTGC |

**Supplementary References**

Chhatre, V. E. & Emerson, K. J. (2017). StrAuto: Automation and parallelization of STRUCTURE analysis. *BMC Bioinformatics 18*: 192.

Earl, D. A. & vonHoldt, B. M. (2012). STRUCTURE HARVESTER: a website and program for visualizing STRUCTURE output and implementing the Evanno method. *Conservation Genetics Resources 4*: 359–361.

Häkli, K., Østbye, K., Kahilainen, K. K., Amundsen, P., & Præbel, K. (2018). Diversifying selection drives parallel evolution of gill raker number and body size along the speciation continuum of European whitefish. *Ecology and Evolution 8*: 2617–2631. doi: 10.1002/ece3.3876

Jakobsson, M., & Rosenberg, N. A. (2007). CLUMPP: a cluster matching and permutation program for dealing with label switching and multimodality in analysis of population structure. *Bioinformatics 23*: 1801-1806.

Mastretta-Yanes, A., Arrigo, N., Alvarez, N., Jorgensen, T. H., Piñero, D., & Emerson, B. C. (2015). Restriction site-associated DNA sequencing, genotyping error estimation and de novo assembly optimization for population genetic inference. *Molecular Ecology Resources 15*: 28–41. doi: 10.1111/1755-0998.12291

Patton, J. C., Gallaway, B. J., Fechhelm, R. G., & Cronin, M. A. (1997). Genetic variation of microsatellite and mitochondrial DNA markers in broad whitefish (Coregonus nasus) in the Colville and Sagavanirktok rivers in northern Alaska. *Canadian Journal of Fisheries and Aquatic Science 54*: 1548–1556.

Peterson, B. K., Weber, J. N., Kay, E. H., Fisher, H. S., & Hoekstra, H. E. (2012). Double Digest RADseq: An Inexpensive Method for De Novo SNP Discovery and Genotyping in Model and Non-Model Species. *PLoS ONE 7*: e37135. doi: 10.1371/journal.pone.0037135

Praebel, K., Westgaard, J.-I., Amundsen, P.-A., Siwertsson, A., Knudsen, R., Kahilainen, K. K., & Fevolden, S.-E. (2013). A diagnostic tool for efficient analysis of the population structure, hybridization and conservation status of European whitefish (Coregonus lavaretus (L.)) and vendace (C. albula (L.)). *Advances in Limnology 64*: 247–255. doi: 10.1127/1612-166x/2013/0064-0026

Turgeon, J., Estoup, A. & Bernatchez, L. (1999). Species flock in the North American Great Lakes: Molecular ecology of Lake Nipigon Ciscoes (Teleostei: Coregonidae: Coregonus). *Evolutio, 53*: 1857–1871. doi: 10.1111/j.1558-5646.1999.tb04568.x

Rogers, S. M., Marchand, M., & Bernatchez, L. (2004). Isolation, characterization and cross-salmonid amplification of 31 microsatellite loci in the lake whitefish (Coregonus clupeaformis, Mitchill). *Molecular Ecology Notes 4*: 89–92. doi: 10.1046/j.1471-8286.2003.00578.x

**Supplementary Files**

**Supplementary Table S1.** ***Proteocephalus fallax* mean infection intensity per fish species in each lake.** Mean intensity is a measure of population size that corresponds to the mean number of worms per infected fish. S.E., standard error.

| **Lake** | **European whitefish species/ecotype** | | **Mean Intensity** | | **S.E.** | | **Intensity range** | |  |
| --- | --- | --- | --- | --- | --- | --- | --- | --- | --- |
| **Bienne** |  | | | **234** | | **52** | |  | |
|  | *C. confusus* | | | 234 | | 52 | | 3-1000 | |
| **Brienz** |  | | | **93** | | **10** | |  | |
|  | *C. albellus* | | | 94 | | 17 | | 8-400 | |
|  | *C. alpinus* | | | 19 | | 7 | | 1-60 | |
|  | *C. brienzii* | | | 121 | | 42 | | 10-749 | |
|  | *C. fatioi* | | | 102 | | 14 | | 7-431 | |
| **Thun** |  | | | **68** | | **8** | |  | |
|  | *C. acrinasus* | | | 56 | | 10 | | 1-216 | |
|  | *C. albellus* | | | 139 | | 23 | | 3-450 | |
|  | *C. alpinus* | | | 6 | | 5 | | 1-10 | |
|  | *C. fatioi* | | | 256 | | 72 | | 52-520 | |
|  | *C. profundus* | | | 18 | | 4 | | 1-130 | |
|  | *C. steinmanni* | | | 34 | | 12 | | 1-230 | |
| **Walen** |  | | | **52** | | **7** | |  | |
|  | *C. duplex* | | | 29 | | 4 | | 1-80 | |
|  | *C. heglingus* | | | 73 | | 13 | | 1-300 | |
| **Langfjordvatn** | | | | **20** | | **4** | |  | |
|  | Profundal | | | 6 | | 3 | | 1-20 | |
|  | Littoral | | | 15 | | 2 | | 1-71 | |
|  | Pelagic | | | 33 | | 10 | | 1-255 | |
| **Suohpatjávri** | |  | | **68** | | **10** | |  | |
|  | Littoral | | | 26 | | 10 | | 1-429 | |
|  | Pelagic | | | 141 | | 14 | | 15-320 | |

**Supplementary Table S2. Number of reads and SNP sets for *Proteocephalus fallax.***

(separate excel file)

**Supplementary Table S3. Bin values for the perialpine and subarctic European whitefish for the retained loci.** (separate excel file)

**Supplementary Figure S1. Environmental correlations with indexes of genetic diversity.** Correlations between lake characteristics and genome diversity measures. The variables surface area (Km^2^), maximum depth (m) and oxygenated depth (m) versus allelic richness (Ar) and gene diversity (Hs), observed heterozygosity (Ho) and inbreeding coefficient (F_IS_). Circles coloured according to the lakes: Suohpatjávri, blue; Langfjordvatn, yellow; Bienne, orange; Walen, pink; Thun, dark orange; Brienz, dark green. Spearman Rank correlation coefficient (R) and *p*-values provided in the graphs. Regression lines were only drawn when the test was significant.

**Supplementary Figure S2. Diversity correlations with parasite sample sizes and SNPs.** Correlations between allelic richness and genetic diversity with *Proteocephalus fallax* sample size from each lake and number of SNPs recovered. Circles coloured according to the lakes: Suohpatjávri, blue; Langfjordvatn, yellow; Bienne, orange; Walen, pink; Thun, dark orange; Brienz, dark green. All correlations were non-significant.
